# Comparing the eating out experiences of consumers seeking to avoid different food allergens

**DOI:** 10.1186/s12889-018-6117-y

**Published:** 2018-11-15

**Authors:** Julie Barnett, Fiona M. Begen, M. Hazel Gowland, Jane S. Lucas

**Affiliations:** 1 Department of Psychology, University of Bath, Claverton Down, Bath, BA2 7AY UK; 2 Allergy Action, St Albans, UK; 3 Clinical & Experimental Sciences, Faculty of Medicine, University of Southampton, Southampton, UK

**Keywords:** Food allergy, Food intolerance, Allergen avoidance, Eating out, Information provision, Gluten, Peanuts / tree nuts, Milk

## Abstract

**Background:**

Eating outside the home is challenging for consumers with food allergy (FA) and intolerance (FI) and lack of allergen information provision in eating out venues can lead to unnecessary restrictions. Following European legislation (2014) designed to improve allergen information provision, little is known about differences in information provision experienced by consumers seeking to avoid particular allergens, or how this impacts on their eating out experiences. This study compared the information provision that consumers with FA/FI to different allergens experience when eating out.

**Methods:**

Using mixed methods, participants were recruited from across the UK and took part in self-report surveys or in-depth interviews. Surveys were completed by 232 participants avoiding either gluten (*n* = 66), nuts (peanuts/tree nuts) (*n* = 94), or milk (*n* = 74), and responses were subject to quantitative analyses. Interviews were carried out with 49 participants avoiding either gluten (*n* = 13), nuts (*n* = 14), milk (*n* = 13) or a combination of these allergens (*n* = 9), and analysed using the framework approach.

**Results:**

Although general improvements in information provision following the legislation were reported, variations in provision between allergen groups led participants seeking to avoid milk to conclude that their dietary needs were less well-understood and seen as less important. These perceptions were reflected in a reluctance to involve eating out venue staff in deliberations about the potential for milk-free meal options.

**Conclusions:**

The provision of visual indicators of the presence of milk and of staff trained in allergen-awareness would improve the eating out experiences of consumers seeking to avoid milk. Medical professions can play a key role in encouraging these patients to pursue their right to make enquiries about allergens in order to avoid accidental milk ingestion when eating out.

**Electronic supplementary material:**

The online version of this article (10.1186/s12889-018-6117-y) contains supplementary material, which is available to authorized users.

## Background

Allergen avoidance is a key management strategy for food allergic (FA) and food intolerant (FI) individuals, and eating outside the home represents a particular risk of accidental allergen ingestion [1] where the provision of information regarding ingredients and food preparation is inadequate or insufficient [2]. Food allergies are caused by an abnormal immunological response to a food, whereas food intolerances have a non-immunological basis [3, 4]. As a general rule, allergic reactions occur very rapidly after ingestion and sometimes lead to immediately life threatening symptoms [5], whilst food intolerances have a delayed reaction and extremely rarely have life threatening symptoms although, like FA, they too can result in significant ill health and impaired quality of life [6]. Between 21 and 31% of accidental allergen ingestions occur when eating in restaurants, and 13–23% occur in other eating out settings such as the work-place or school canteens [7]. In cases of children suffering anaphylaxis to a known food allergen, over half of these occurred outside the home [8].

EU legislation (EU Food Information for Consumer Regulation No. 1169/2011, (EU FIC)) introduced in December 2014 [9], requires food businesses providing and selling non-prepacked foods to provide allergen information relating to the inclusion of any of 14 specified food allergens (peanuts, tree nuts, milk, soya, mustard, lupin, eggs, fish, molluscs, crustaceans, cereals containing gluten, sesame seeds, celery, and sulphur dioxide at levels above 10 mg/kg, or 10 mg/litre) as ingredients in their foods. The legislation thus affects restaurants, takeaway establishments, food stalls, institutions including prisons and nursing homes, as well as workplace and school canteens. Allergen information can be provided in written or verbal form. Where verbal information is provided, written information must also be available to customers within the venue. Thus far however, there has been little consideration of how people’s eating out experiences - including the provision of allergen information – varies in relation to different allergens.

Given that adverse reactions can occur in response to any of these allergens, differences in the quality of information provided about them is important. Little work has considered the differential impact of seeking to avoid particular allergens or how experiences of seeking to avoid particular allergens vary. Although adherence to an allergen-free diet has been associated with poorer quality of life, and significant social and behavioural restrictions [10–14], literature tends to generalise across populations avoiding allergens [11, 15] or focus on one specific allergen grouping; most commonly avoidance of peanuts and/or tree nuts [16–18], or gluten in coeliac populations [19, 20]. Where studies have focused on the difficulties encountered by populations seeking to avoid ‘staple food’ allergens (milk, wheat, eggs) [21–23], no distinction has been made between allergens in order to assess any differences experienced between these groups. Where differences between allergen avoidance groups have been considered in parents of FA children, there was greater psychosocial impact on parents seeking to avoid milk or eggs on behalf of their child than for parents seeking to avoid other food allergens [24, 25].

As yet, the eating out experiences of populations seeking to avoid particular allergens has not been considered. In light of the EU FIC legislation, eating out venues are required to provide information about the content of each of the 14 allergens in their foods, and attention has recently turned to the adequacy of this information provision for each allergen. For example, online resources such as ‘Guide to eating out with a food allergy’ [26], show how well some eating out venues cater for customers avoiding a particular allergen by reporting the availability of allergen-free meals for each of the 14 allergens.

Evaluating the impact of the EU FIC legislation provided the opportunity to compare the information provision that customers with FA/FI experience in relation to different allergens when eating out. In order to investigate this, in a mixed methods study we conducted semi-structured interviews and self-report surveys with customers who avoided particular allergens (gluten, nuts: peanuts/tree nuts, or milk) following implementation of the legislation. We assessed differences between these groups based on their satisfaction with allergen information provision, and their preferences for written and verbal forms of information delivery.

## Methods

### Overview

As part of wider programme of longitudinal research into the eating out experiences of adults and parents/carers of children with FA/FI [27] prior to (2014) and following (2016) implementation of EU FIC legislation [28], we recruited participants from across the UK to take part in either (A) In-depth interviews in 2014 and 2016, or (B) Surveys in 2014 and/or 2016. Ethical approval was gained from the institution’s departmental ethics committee prior to recruitment (Ref: 14–055/16–146). The current paper reports findings relating to participants who reported avoiding gluten, nuts (peanuts and/or tree nuts) or milk in 2016 interviews or surveys. Interview findings from 2014 are reported elsewhere [2].

### Online survey

#### Recruitment and study population

Survey participants were recruited from across the UK by a professional market research agency: Acumen Fieldwork-Medical (66%) and using the websites and mailing lists of three UK-based charities: Allergy UK (28%), Anaphylaxis Campaign (3%), Coeliac UK (3%). Between November and December 2016, 392 participants completed the survey. Of these, 188 (48%) had been recruited to complete a prior version of the survey in 2014 and returned to complete the 2016 survey, and 204 (52%) were recruited as new participants to complete the 2016 survey. Of the total 2016 survey population, 232 (59%) participants were included in analyses because they avoided either gluten, nuts or milk when eating out.

#### Online survey

Participants completed a screening questionnaire to ensure that they met the minimum requirements for inclusion in the study. The inclusion criteria were that participants aged over 18, or their child in the case of parents/caregivers: a) experienced reactions to one or more of the 14 allergens covered by the EU FIC legislation; b) ate out at, or ordered takeaway food from a restaurant, café, coffee shop, fast food outlet, or any other place where they can buy non-prepacked food; c) sought to avoid one or more of the 14 allergens covered by the EU FIC legislation when eating out or ordering takeaway food; d) experienced one or more symptoms typically associated with IgE-mediated food allergy or non-IgE-mediated reactions (classified as food intolerance in this study). Survey results for participants seeking to avoid nuts (tree nuts and peanuts), gluten or milk are reported. Classification criteria are shown in Table 1.

**Table 1:** Allergy or intolerance classification criteria and allergy severity classification criteria

Classification	Symptoms	Severity
ALLERGY: Symptoms associated with IgE-mediated reactions	‘Stinging nettle’ rash, urticaria, hives, Itching or swelling of the lips, tongue or mouth, asthma, wheezing, facial swelling (does not experience ‘severe’ symptoms)	MILD/MODERATE *(Does not include ‘severe’ symptoms)*
	Breathing difficulties, anaphylaxis, collapse *(May additionally include symptoms associated with non-IgE-mediated reactions)*	SEVERE *(May additionally include ‘mild/moderate’ symptoms)*
INTOLERANCE: Symptoms associated with non-IgE-mediated reactions	Vomiting, Diarrhoea, Sneezing, Catarrh, Hyperactivity, Tiredness, Stomach cramps, Other digestive problems (e.g. bloating, constipation), Eczema flare, Migraines/headaches, Aching joints/muscles, Behavioural/mood changes *(Does not include symptoms associated with IgE-mediated reactions)*	

#### Survey content

We designed an online survey relating to attitudes and behaviours when eating out specifically for the study. Survey design was informed by a literature review, discussions with support groups and interviews conducted in 2014, prior to EU FIC legislation [2]. Interviews were coded and analysed using the framework approach. Themes derived from these interviews were used as the basis for survey items, which were worded and sense-checked by the research team before being piloted with a small sample (*n* = 20) of participants. Survey subscales included: ‘Reliance on speaking to staff’; ‘Satisfaction with written information’; ‘Staff as an additional information source’; ‘Preference for separate allergen menu’; and two single items-‘Menu invites you to ask staff’ and ‘Sign invites you to ask staff’. All 2014 survey items were retained in the 2016 survey. Full details of subscale items and item reversals are shown in Table 2.

**Table 2:** Details of survey subscales

Survey subscale	Survey item^a^ (R) = Reverse scored	Response scale	Cronbach’s alpha
Reliance on speaking to staff	- I am happy to ask serving staff about allergens in the food they are serving - I ask to speak to the manager if I want more information about allergens in the dishes - I ask to speak to the chef if I want more information about the meal being cooked for me - I don’t like asking staff questions about allergens (R) - I feel awkward and embarrassed to ask staff questions about the food they are serving (R)	0 ➔ 6 Never ➔ Always	.685
Satisfaction with written information	- Menu information online (R) - The menu displayed outside the place (R) - The menu displayed at the counter (R) - The menu at the Table (R) - Phone apps (R) - Information folder about ingredients of foods being served (R)	1 ➔ 5 Very satisfied ➔ Very dissatisfied	.827
Staff as an additional information source	- Even if there was information about allergens on the menu I would like to ask a member of staff about the dish (R) - No matter how good the written information is I would prefer to talk to staff (R)	1 ➔ 5 Strongly agree ➔ Strongly disagree	.834
Preference for a separate allergen menu	- I would like to see separate menus for people with particular food intolerance or allergies. (R) - I want to know from the menu how the food is cooked not just what is in it (R) - It is reasonable to expect that there are separate menus to help people avoid particular allergens (R)	1 ➔ 5 Strongly agree ➔ Strongly disagree	.730
Menu invites you to ask staff about allergens	- I like it when it says in the menu that they welcome customers with allergies and intolerances asking about dishes (R)	1 ➔ 5 Strongly agree ➔ Strongly disagree	Single item
Sign invites you to ask staff about allergens	- I like it when there is a sign up that says that they welcome customers with allergies and intolerances asking about dishes (R)	1 ➔ 5 Strongly agree ➔ Strongly disagree	Single item

^a^Survey items were not subject to factor analysis

#### Procedure

Following provision of informed consent, participants meeting the inclusion criteria were routed to the survey for completion.

#### Data analyses

Statistical analyses were carried out using IBM SPSS Statistics (v22). Data was screened to ensure no violation of the assumptions of normality, linearity, and homoscedasticity. The extent of missing data (less than 2%) and non-response patterns were assessed to see if missing items would impact on analyses (Little’s MCAR test (*p* > .05)). Missing values for items within subscales were imputed using expectation-maximization (EM) [29] and subscale reliabilities were calculated. Differences between allergen groups (gluten, nuts or milk) were analysed using mixed ANOVAs including ‘Adult/Parent’, ‘food allergy/intolerance’ as independent variables (IVs), and the four eating out subscales and two single-item questions as outcome variables. Post hoc analyses was carried out using Bonferroni procedure. A post hoc cut-off of *p* ≤ .05 was used, although post hoc tests approaching significance (*p*=. 051 – *p*=. 056) are also reported.

### In-depth interviews

#### Recruitment and population

Full details of 2014 interview recruitment procedure, populations and results are reported elsewhere [2]. Of the 57 participants who completed interviews between June and July 2016, all had been recruited through a professional research agency (as above) and had completed previous interviews in 2014. Of the total interview population in 2016, 49 (86%) participants were included in analyses because they avoided gluten, nuts and/or milk when eating out.

#### Procedure

In-depth semi-structured interviews were carried out in participants’ homes following an interview protocol detailing questions and possible prompts (a copy of this interview protocol can be provided on request from the corresponding author). Each interview was audio-recorded with participants’ permission. Initial questions related to any changes that had occurred in returning participants’ lives; and in relation to their food allergy in particular. The interview then focused on participants’ recent eating out experiences and any changes in these, including their encounters with information about food allergens. They were asked for their reflections and evaluations of these changes, and about the impact of the legislation on allergen information provision in relation to their eating out experiences. Interviews lasted between 27 and 76 min.

#### Analyses

Interview recordings were transcribed verbatim and explored in detail using framework analysis [30]. Interviews were coded and analysed using QSR-NVivo (version 10). Identified themes are illustrated in results. In order to maintain anonymity, participant details are indicated in brackets as follows: A/P refers to Adult/Parent; participant number; and reported food allergens. Italicised text within quotes reflects interviewer prompts.

## Results

### Online survey

Characteristics of survey participants are shown in Table 3 (further demographic details are shown in Additional file 1). Of the 392 participants who completed surveys, 232 (59%) avoided one of the target food allergens, either: gluten, nuts or milk. Participants who avoided more than one target food allergen (*n* = 121, 31%) and those who avoided an allergen other than gluten, nuts or milk (*n* = 39, 10%) were excluded from analyses.

**Table 3:** Characteristics of survey population based on allergen avoided

Variable	Gluten (*n* = 66) n (%) or M (SD)	Nuts (*n* = 94) n (%) or M (SD)	Milk (*n* = 72) n (%) or M (SD)
Adult	56 (84.8)	40 (42.6)	39 (54.2)
Parent	10 (15.2)	54 (57.4)	33 (45.8)
Gender			
Adult/Parent			
Male	13 (19.7)	12 (12.8)	9 (12.5)
Female	53 (80.3)	81 (86.2)	61 (84.7)
Child^a^			
Male	4 (40.0)	32 (59.3)	19 (57.6)
Female	6 (60.0)	21 (38.9)	14 (42.4)
Age (yrs)			
Adult/Parent	41.2 (11.9)	39.6 (9.7)	37.6 (10.5)
Child	8.5 (3.8)	10.6 (4.2)	5.1 (3.8)
Food allergic	8 (12.1)	86 (91.5)	27 (37.5)
Food intolerant	58 (87.9)	8 (8.5)	45 (62.5)
Diagnosis			
Clinical diagnosis *(by GP; Dietician or Allergy specialist at hospital)*	47 (71.2)	84 (89.4)	44 (61.1)
Self diagnosis	19 (28.8)	10 (10.6)	28 (38.9)
Severity of reaction (FA only)^b^			
Mild/Moderate	5 (62.5)	28 (32.6)	23 (85.2)
Severe	3 (37.5)	58 (67.4)	4 (14.8)
Time since diagnosis (yrs)			
< 2	12 (18.2)	3 (3.2)	14 (19.2)
2–4	20 (30.3)	23 (24.5)	24 (33.3)
5–9	18 (27.3)	24 (25.5)	22 (30.6)
≥ 10	16 (24.2)	43 (45.7)	11 (15.3)
Treatment			
Avoidance	66 (100)	94 (100)	72 (100)
Antihistamines	4 (6.4)	67 (71.3)	15 (20.8)
Injectable adrenaline	1 (1.5)	66 (70.2)	2 (2.8)
Inhaler	1 (1.5)	33 (35.1)	10 (13.9)
Special diet	32 (48.5)	9 (9.6)	25 (34.7)
Support group membership	27 (40.9)	36 (38.3)	7 (9.7)

^a^Child % calculation based on total parent participants per allergen group

^b^Severity % calculation based on total FA participants per allergen group

Where % total < 100, there are missing values. Where % total > 100, participants could select multiple responses

Summarised in Table 4, the survey revealed significant differences in participants’ perceptions of information provision depending on whether they wished to avoid gluten, nuts or milk when eating out. Unless otherwise stated, there were no interactions between ‘allergen avoided’ and other IVs (‘Food allergy/Food intolerance’ or ‘Adult/Parent’) (all ps > .05).

**Table 4:** Differences in perceptions of information provision for participants avoiding Gluten, Nuts and Milk following legislation^a^

Survey subscale	Gluten	Nuts	Milk				
		Mean (SD)		df	F	η_p_^2^	p
Reliance on speaking to staff	3.26 (1.25)	3.79 (1.27)	3.15 (1.22)	2, 220	4.20	.037	.016
Satisfaction with written information	3.30 (0.91)	3.59 (0.73)	3.41 (0.73)	2, 220	3.13	.028	.046
Staff as an additional information source	3.48 (1.29)	4.00 (1.02)	3.10 (1.23)	2, 220	4.13	.036	.017
Preference for separate allergen menu	4.11 (0.87)	3.93 (0.97)	3.49 (0.99)	2, 219	5.15	.045	.007
Menu invites you to ask staff about allergens	4.55 (0.79)	4.67 (0.67)	4.33 (0.87)	2, 218	3.53	.031	.031
Sign invites you to ask staff about allergens	4.59 (0.78)	4.60 (0.81)	4.29 (0.94)	2, 217	3.83	.034	.023

^a^Higher mean score indicates greater levels of agreement

#### Reliance on speaking to staff

There was a significant main effect of ‘allergen’ (gluten/nuts/milk) on participants’ reliance on speaking to staff (*p* < .05). Participants avoiding nuts reported a greater reliance on speaking to staff than those avoiding gluten (*p* = .019), and those avoiding milk (*p* = .003).

#### Satisfaction with written information

There was a significant main effect of ‘allergen’ (gluten/nuts/milk) on participants’ satisfaction with written information (*p* < .05). Post hoc analysis approached significance (*p* = .053) suggesting that those who avoided nuts were more satisfied that written information could aid confident food choices than those avoiding gluten.

#### Staff as an additional information source

There was a significant main effect of ‘allergen’ (gluten/nuts/milk) on participants’ preference for staff as an additional information source (*p* < .05). Participants avoiding nuts (*p* = .009) and those avoiding gluten (*p* = .001) both preferred staff as an additional source of information in comparison to those avoiding milk.

#### Preference for separate allergen menu

There was a significant main effect of ‘allergen’ (gluten/nuts/milk) on participants’ preference for a separate allergen menu (*p* < .01). Participants avoiding nuts (*p* = .007) and those avoiding gluten (*p* = .001) had greater preference for a separate allergen menu as a potential source of information than those avoiding milk.

#### Menu invites you to ask staff

There was a significant main effect of ‘allergen’ (gluten/nuts/milk) on participants’ perceptions of a statement on the menu inviting customers to ask staff about dishes (*p* < .05). Participants avoiding nuts were more positive about the menu inviting customers to ask about dishes than those avoiding milk (*p* = .016).

#### Sign invites you to ask staff

There was a significant main effect of ‘allergen’ (gluten/nuts/milk) on participants’ perceptions of a sign inviting customers to ask staff about dishes (*p* < .05). Post hoc analysis approached significance (*p* = .056) suggesting that those avoiding nuts were more positive about the sign inviting customers to ask about dishes than those avoiding milk.

### In-depth interviews

Characteristics of interview participants are shown in Table 5. Of the 57 participants who completed interviews in 2016, 49 (86%) avoided gluten, nuts and/or milk. Participants who avoided an allergen other than gluten, nuts or milk (*n* = 8, 14%) were excluded from analyses.

**Table 5:** Characteristics of interview population based on allergen avoided

Variable	Gluten (*n* = 13) n (%) or M (SD)	Nuts (*n* = 14) n (%) or M (SD)	Milk (*n* = 13) n (%) or M (SD)	Multiple^a^ (*n* = 9) n (%) or M (SD)
Adult	12 (92.3)	11 (78.6)	8 (61.5)	9 (100)
Parent	1 (7.7)	3 (21.4)	5 (38.5)	0
Gender				
Adult/Parent				
Male	1 (7.7)	5 (34.7)	4 (30.8)	1 (11.1)
Female	12 (92.3)	9 (64.3)	9 (69.2)	8 (88.9)
Child^a^				
Male	0	2 (66.7)	2 (40.0)	0
Female	1 (100)	1 (33.3)	3 (60.0)	0
Age (yrs)				
Adult/Parent	37.5 (18.0)	38.9 (15.2)	43.1 (10.8)	39.78 (13.3)
Child^b^ < 10	0	0	1 (20.0)	0
10–14	0	2 (66.7)	0	0
> 14	1 (100)	1 (33.3)	4 (80.0)	0
Food allergic	1 (7.7)	14 (100)	2 (15.4)	5 (55.6)
Food intolerant	12 (92.3)	0	11 (84.6)	4 (44.4)
Diagnosis				
Clinical diagnosis * (by GP; Dietician or Allergy specialist at hospital)*	10 (76.9)	13 (92.9)	7 (53.8)	3
Self diagnosis	3 (23.1)	1 (7.1)	6 (46.2)	2
Severity of reaction (FA only)^c^				
Mild/Moderate	1 (100)	7 (50.0)	2 (100)	3 (60.0)
Severe	0	7 (50.0)	0	2 (40.0)
Time since diagnosis (yrs)				
< 3	0	1 (7.1)	0	0
3–7	8 (61.5)	3 (21.4)	5 (38.5)	4 (44.4)
≥ 7	5 (38.5)	10 (71.4)	8 (61.5)	1 (11.1)
Treatment				
Avoidance	13 (100)	14 (100)	13 (100)	9 (100)
Antihistamines	2 (15.4)	7 (50.0)	3 (23.1)	3 (33.3)
Injectable adrenaline	1 (7.7)	7 (50.0)	0	1 (11.1)
Inhaler	0	4 (28.6)	0	0
Special diet	8 (61.5)	2 (14.3)	5 (38.5)	1 (11.1)
Support group membership	2 (15.4)	3 (21.4)	0	2 (22.2)

^a^Two or more target allergens avoided- e.g. gluten and milk

^b^Child % calculation based on total parent participants per allergen group

^c^Severity % calculation based on total FA participants per allergen group

Where % total > 100, participants could select multiple responses. Where % total < 100, there are missing values

Following implementation of the legislation, three overall themes were described by participants in relation to their observations and experiences of allergen information provision when eating out. Participant responses focused on management of their FA/FI when eating out and related to: ‘disparities in allergen information provision’, ‘understanding the needs of customers avoiding different allergens’, and ‘customer demand for information about specific allergens’.

### Disparities in allergen information provision

Following implementation EU FIC, the majority of participants had observed general improvements in the provision of allergen information when eating out; though they noted that these improvements were largely focused on the provision of information for customers seeking to avoid nuts or gluten. For many participants, a disparity in allergen-specific information was observed, regardless of the allergen that they themselves sought to avoid (Table 6: quote 1).

**Table 6:** Disparities in the provision of allergen information

1)	‘I’ve definitely seen it [allergen information] a lot more about. It’s kinda really visible in a lot of places which is alright… I think it is just a few of them [allergens]… Just nuts and gluten.’ (A32, FI: Gluten)
2)	‘… they have an entirely separate menu so I feel very comfortable going there… my preference is a separate gluten free menu but I realise it’s probably unrealistic to expect everywhere to do that so I guess, if I’m going into a place I know doesn’t have a gluten free menu it just makes things 10 times easier if they’ve got a little symbol… the little symbols and then a key under every dish. Just printed those symbols and then it’s done, easy.’ (A13, FI: Gluten)
3)	‘… going out it normally says on the menu now. It will have a little ‘N’ next to it or something... *Is that a new thing?* It’s getting better since I last saw you. Most places do it now and they do it for gluten free and things… It will say if it’s got nuts in. It makes it easier for them because they’re not having to answer your questions all the time. You can read the menu and say “that has got nuts in”.’ (A58, FA: Peanuts & tree nuts)
4)	‘In my experience it’s [the legislation] been ineffective for his condition and I have actually been in a restaurant with a friend that was presented with a gluten free menu for breakfast and I was so impressed that they could do that with the gluten free but [it] wasn’t available for dairy- and in fact the same restaurant was able to present a different menu for [healthy weight loss] diets which I thought was amazing- that they could go to that effort but yet it wasn’t available for something that seems to effect a lot of people.’ (P7, FI: Milk)
5)	‘… mainly vegetarian and vegan, yep, and gluten free and they were the main ones. *But not dairy?* Not dairy, nothing. I haven’t come across a single place that talks about dairy free. But I think it’s because it’s not very well understood.’ (A51, FI: Milk)
6)	‘On the menu where you see the ‘V’ or the ‘G’ and all that business, to have a ‘D’ for dairy so that covers any type of dairy then at least you could say, actually for me, I would rule it all out…’ (A44, FI: Milk)

For participants seeking to avoid gluten, the separate ‘gluten-free’ menu was seen as a gold standard which was becoming increasingly available. In the absence of this provision, the use of a symbol or letter displayed beside each dish on the main menu served as a simple and trusted indicator which facilitated food choices (Table 6: quote 2). Similarly, for participants seeking to avoid nuts, the display of a symbol or letter ‘N’ beside menu items had become widespread, and enabled them to make independent food choices without the need to involve staff in their decision-making process. (Table 6: quote 3).

Participants seeking to avoid milk had also observed the improvements in information provision for those avoiding nuts or gluten, but had not seen similar improvements in relation to their own dietary needs. These participants were impressed by the gluten-free provision that was now available, and wished that similar information was available for milk-free diets (Table 6: quote 4). They also noted that diets which might be deemed ‘lifestyle choices’ were also catered for, whilst their need for information about the milk content of foods remained largely neglected and misunderstood (Table 6: quotes 4 & 5); a scenario that they felt could be resolved with little effort on the part of eating out venues (Table 6: quote 6).

### Understanding the needs of customers avoiding different allergens

Many participants seeking to avoid milk felt that their dietary needs were not well understood, and that this in turn might be leading to a lack of appropriate allergen information provision in eating out environments. Participants noted that many eating out staff failed to understand their need for avoidance of milk as a ‘hidden ingredient’ within many dishes. In the absence of tangible written allergen information, participants used subtle social cues to detect misunderstanding on the part of venue staff (Table 7: quote 1), and often interpreted these cues as a more generalised indicator that their needs were underestimated or undervalued (Table 7: quote 2).

**Table 7:** Understanding the needs of customers avoiding different allergen

1)	‘… there’s so many different things it [milk] could be in so that’s why I think people who work in restaurants and cafes they just sort of panic and don’t fully understand… they just assume dairy for me is cheese or butter or it’s got cream on it. Well no, it’s not the cream I’m talking about, I’m talking about the content in the scone for example or in the cake.’ (A44, FA: Milk)
2)	‘I just feel that actually some people don’t feel like it’s actually worthy of a restaurant going out of your way for it. I still don’t feel comfortable, I still don’t think [milk allergy/intolerance is] an acceptable thing to legitimately have. *You think there’s a stigma attached?* Yeah, I still think people have.’ (A51, FI: Milk)
3)	‘When I used to say coeliac or gluten free they would look at you a bit… now I think staff are more totally up on it. So I think in the majority of places they are told about it, I mean obviously there is nut allergies and things, but nut allergy is quite obvious, it’s nuts. When you say gluten they think “well” you know “what’s that in?”… unless you’ve come across it, I would have been the same. But I have found it much better.’ (A57, FI: Gluten)
4)	‘… things have changed and got better yet I’ve still had reactions- and of course with the growing increase of “fad diets” there is always the risk that you’re not taken seriously and you know, yeah great, “gluten free” is getting awareness these days but it’s about whether it’s the “right type”, or whether people just think it’s… you have to be taken quite seriously as a coeliac sufferer and I don’t think we are anymore. So it’s kind of swings and roundabouts.’ (A13, FI: Gluten)
5)	‘I think what the problem is particularly with the nuts is that there are so many things made without nuts and get cross contaminated, so they tend to see it as not as problematic as someone who is coeliac. I think they look at it as “oh you’ve got a nut allergy”, yes. I think there are places that don’t tend to think it’s serious.’ (P2, FA: Peanuts, Tree nuts)

For participants seeking to avoid gluten, the issue of gluten as a ‘hidden ingredient’ coupled with indictors of confusion exhibited by venue staff had been experienced in the past, and were now less common in light of increased staff awareness and improved information provision (Table 7: quote 3). These improvements, whilst welcomed, did not guarantee a gluten-free eating out experience however. A minority of participants expressed concern that the popularity of gluten-free diets as a ‘lifestyle choice’ had undermined staff perceptions of the importance of gluten avoidance for those with the medical need to remain gluten-free (Table 7: quote 4). Similarly for those seeking to avoid nuts, whilst improvements in information provision were appreciated, the risk of cross-contamination and potential for staff underestimation of that risk undermined their confidence in ensuring an nut-free eating out experience (Table 7: quote 5).

### Customer demand for information about specific allergens

One participant, who worked in an eating out venue, provided insights into the relative frequency of customer enquiries about the allergens. They noted that such enquiries were infrequent; relating to the gluten and nuts, but not to the milk content of foods (Table 8: quote 1). Participants who sought to avoid milk speculated that their own lack of communication with staff might imply to food businesses that there was little demand for the provision of milk-related allergen information (Table 8: quote 2 & 3). Participants compared the relative impact of their milk-related symptoms with those who experience life-threatening reactions to nuts. Whilst they wished that eating out venues could appreciate the discomfort that they experienced due to accidental allergen consumption (Table 8: quote 4 & 5), they equally tended to underplay such reactions and often failed to inform the eating out venue that a problem had occurred.

**Table 8:** Customer demand for information about specific allergens

1)	‘*… out of interest what are the sort of allergies and intolerances that you hear more of, most of?* Gluten, nuts and seafood. *Okay right and very often?* No, not often at all actually. Like a lot, gluten more than anything... Nuts maybe four or five times in a year, yeah not often at all. I don’t know whether it’s not that common, or people just don’t mention it and seafood maybe once or twice a year to be fair, not often at all.’ (A59, FA: Peanuts, Tree nuts)
2)	‘… I think there aren’t enough people who are lactose intolerant for companies to see it as viable. Or enough people to make a fuss. So I’m part of the problem I think. There aren’t enough people making a fuss about it because of people not wanting to make it a big thing so companies don’t have to make a big deal about it, but if everyone who had slight lactose intolerance… pushed in restaurants I think there would be a bigger appeal for it. We are part of the problem.’ (A51, FI: Milk)
3)	‘Well, if everywhere could do soya milk that would be excellent, or start having optional lactose or dairy free cheese as options rather than having to not have anything that’s a milk product but I don’t know if there’s an economic imperative for shops. If there’d be enough customers who would be interested in that, there might be. There might be plenty of people who are just avoiding these things who would buy them if they knew that they could have nachos with lactose free cheddar, then they would but I suppose until they try that they don’t know.’ (A10, FI: Milk)
4)	‘Nut allergies I think prevail a lot. I think they are aware of nut allergies… I don’t think they think anything else is… it’s a killer, do you know what I mean? But I’m not going to die eating a sandwich, but I can be in pain for hours and it can have a massive effect, because you can’t do anything.’ (A34, FI: Gluten, Milk)
5)	‘… it’s not life threatening like if I had nut [allergy] or anything like that. So, I just know that night I’m going to suffer… I think if I was nut intolerant I would be very… but because it’s not life threatening I think I tend to put up with it and think “I won’t have that again”.’ (A45, FA: Milk)

## Discussion

Using a mixed methods approach, this study indicates that the eating out experiences of consumers with FA/FI differ depending on the food allergen that they are seeking to avoid, and that allergen-based inequities in information provision are impacting on some consumers with FA/FI following the introduction of EU FIC legislation in December 2014. Specifically, not only do those avoiding milk have less positive experiences, but in addition they perceive that the provision made for those avoiding other allergens tends to be better. Participants seeking to avoid milk had also observed the improvements in information provision for those avoiding nuts or gluten, but had not seen similar improvements in relation to their own dietary needs. They noted that many staff in eating out venues failed to understand their need for avoidance of milk as a ‘hidden ingredient’ within many dishes.

In general, survey participants reported being moderately satisfied with the availability and adequacy of allergen information provision when eating out, and interview participants suggested that this provision was an improvement on the allergen information made available prior to EU FIC. However, satisfaction levels and perceived improvements in provision differed depending on whether participants sought to avoid gluten, nuts, or milk as ingredients in foods. Amongst survey participants, those seeking to avoid milk were less satisfied with the information provided for their specific dietary needs in comparison to participants avoiding nuts, and to a lesser degree those avoiding gluten. In particular, they were less likely to involve staff in their deliberations about the potential for allergen free meal options- either by asking staff directly about the allergen content of foods or as an additional information resource following inspection of the menu. They were also less likely to take a positive view of written statements inviting customers to ‘ask staff’. Although this is in part unsurprising given that research prior to the implementation of EU FIC indicated that consumers with FA/FI were often reluctant to make enquiries of staff [2], crucially, such reluctance was similar across allergen groups at this earlier time-point. Prior to the legislation, there were no differences between gluten, nut or milk avoiding participants in relation to their satisfaction with the information provided for specific dietary needs or their likelihood to involve staff in deliberations about allergen-free meal options [27, 31] (see also Additional file 2). Therefore, findings suggest that these differences have arisen since the legislation.

Under the themes ‘disparities in allergen information provision’, ‘understanding the needs of customers avoiding different allergens’, and ‘customer demand for information about specific allergens’, in-depth interviews provided insights into the potential reasoning behind participant survey responses. Whilst participants seeking to avoid gluten and nuts reported improvements in written allergen information provision when eating out, those seeking to avoid milk observed no such improvements. It is likely that this post-legislative disparity between groups created feelings of inequity of provision that did not exist prior to the legislation’s implementation, thus fragmenting allergen avoiding populations. This is an important consideration for eating out venues given that consumers with FA/FI tend to equate the adequacy of allergen information provision with wider judgements about the venue’s ‘understanding’, ‘allergen-awareness’ and ‘capacity’ to accommodate specific dietary needs safely [2]. For participants seeking to avoid milk, an absence of relevant allergen information suggested a lack of understanding on the part of eating out venues and their staff. These participants were less likely to trust staff as an information source, and were potentially less likely to patronise such venues as a result. Furthermore, as noted in previous research consumers with FA/FI attempt to balance their need for allergen avoidance, with their wish to avoid being seen as ‘making a fuss’ and creating ‘misunderstanding’ [32, 33]. For those seeking to avoid milk, insufficient allergen information provision suggested that asking staff might indeed lead to misunderstanding and potential social embarrassment. They were less willing to speak to staff about their dietary requirements, and more likely to expose themselves to the risk of accidental allergen consumption as a consequence.

The perceived understanding of the needs of some consumers with FA/FI (nuts and gluten) in comparison to others (milk), led participants seeking to avoid milk to conclude that the implications of their accidental allergen consumption were taken less seriously, and their concerns seen as less legitimate than other allergen-avoiding groups. This distinction has been observed in FA and FI populations, where FI can be viewed as more ‘socially problematic’ than FA, due to the ambiguity of FI symptoms and diagnosis when compared to FA [34]. Some of our participants who sought to avoid milk due to lactose intolerance perceived that there was ‘stigma’ attached to the condition and recognised that their own reticence in speaking to staff due to concerns about being seen as ‘making a fuss’ might in turn be viewed as a lack of ‘demand’ for milk allergen information provision on the part of eating out venues.

Equally, it is important for eating out venues to consider the implications of accidental allergen consumption for customers with severe FA to milk. Whilst FA to peanuts/tree nuts is more common, and the potential for anaphylaxis amongst this population more widely understood, cow’s milk is the most common cause of anaphylaxis amongst UK children [5] and persistence of milk FA into adulthood is associated with greater risk of severe reactions [35].

### Implications

This study is the first to provide insight into the perceived differences in allergen information provision for particular allergens, and most importantly, the difficulties that consumers with FA/FI report when seeking to avoid milk whilst eating outside the home.

Alongside their legal responsibilities to provide allergen information for consumers as a result of EU FIC, it is important that eating out providers understand that FA/FI customers are sensitive to inequities in allergen information provision and interpret these as a wider indicator of customer care and food safety in venues. Any such inequities are likely to be magnified for FA/FI customers who seek to avoid ‘staple foods’ (milk, wheat, eggs) which are ubiquitous in the western diet and more difficult to avoid as a result [21–23]. An absence of customer enquiries about particular allergens- in this case milk - should not be interpreted as a lack of demand for information about the allergen, and participants felt that venues can usefully convey their willingness and ability to accommodate these customers using simple, visible visual indicators such as letters/symbols on the menu. Increased staff allergen awareness training [36] and effective communication systems between food preparation and serving areas [2] will help to ensure that FA/FI customers feel more confident and secure in their food choices when eating out; regardless of the allergen that they are seeking to avoid. Normalising the notion that customers are able and entitled to make their allergen requirements known may be particularly helpful. For example, serving staff could take a proactive approach at the table, by enquiring as to whether customers have any specific dietary requirements [2]. They should be particularly aware that those seeking to avoid milk may be less confident in the ability of the venue to provide a meal without the presence of this allergen.

Health professionals (allergists, dieticians, general practitioners), support groups and charities have important contributions to make by educating and encouraging their FA/FI patients- and those avoiding milk in particular - to be confident in requesting and expecting the provision of allergen information when eating out, as they are entitled to do since the introduction of EU FIC. Patients can also be encouraged to use proactive techniques such as informing eating out venues in advance [15] or carrying an allergy/coeliac information card [37] in order to ameliorate their fears of embarrassment in the inherently social setting of the eating out environment.

### Limitations

Participants self-reported their FA/FI status, and a minority were self-diagnosed alongside those who reported receiving a clinical diagnosis. Entry to the study was through the careful application of symptom-based FA/FI criteria although we recognise it is unlikely that classification of patients as FA or FI would accord with a medical diagnosis. However, our approach of making the distinction between populations based on the allergen that they were seeking to avoid rather than between FA and FI renders this limitation as less problematic. Our approach allowed us to highlight the common difficulties experienced by milk avoiding FA/FI participants, and these difficulties were particularly salient given that no allergen-based differences between FA and FI populations were shown in analyses. Furthermore, in the context of eating out, the distinction between FA and FI becomes less relevant because the legal requirement for venues to provide allergen information applies for all customers and is not contingent on their FA/FI status.

We also acknowledge that we took a conservative approach in survey analyses. In order to ensure that responses were attributable to each particular allergen avoided, we only included participants who avoided either gluten or nuts or milk and did not include those who reported avoiding multiple allergens. It is likely that participants who sought to avoid multiple allergens experienced greater difficulties when eating out [11]. Lastly, we recognise that we were unable to include other allergens in our analyses due to insufficient participant numbers. It is possible that populations seeking to avoid different allergens- and in particular those seeking to avoid eggs which are also a ‘staple food’ [21–23] - would have reported inequities in allergen information provision akin to those reported for milk allergen in this study. Possible limitations to generalisability of the survey results should be borne in mind in the light of these issues.

## Conclusion

A mixed methods approach was valuable in exploring the experiences of those seeking to avoid gluten, milk and nuts when eating out. Through the application of surveys and interviews, FA/FI participants reported that there were general improvements in allergen information provision in eating out venues following introduction of EU FIC legislation. However, inequities in the provision of allergen information for particular allergens (gluten, nuts, milk) led participants seeking to avoid milk to conclude that their dietary needs were less well-understood and seen as less important. These perceptions were reflected in a reluctance to involve eating out venue staff in deliberations about the potential for allergen free meal options, and limited the food choices of those seeking to avoid milk as a result. The provision of visible visual indicators on menus of the presence of milk and increased allergen-awareness training for staff can play a key role in increasing confidence in the eating out venues and improve the eating out experience of customers seeking to avoid milk. Medical professionals also have a key role to play in educating and encouraging their FA/FI patients to pursue their legal right to make allergen enquiries in order to avoid accidental milk allergen consumption when eating out.

## Additional files

Additional file 1: Further demographic and background characteristics of survey participants, Description of data: Further demographic and background characteristics of survey participants. (DOCX 14 kb)

Additional file 2: Perceptions of information provision for participants avoiding Gluten (*n* = 149), Nuts (*n* = 272) and Milk (*n* = 77) prior to legislation, Description of data: Perceptions of information provision for participants avoiding Gluten (*n* = 149), Nuts (*n* = 272) and Milk (*n* = 77) prior to legislation. (DOCX 14 kb)

## Abbreviations

FA: Food Allergy
FI: Food Intolerance
