# Supplementary material for: Comparing the eating out experiences of consumers seeking to avoid different food allergens
Source: BMC Public Health. 2018 Nov 15;18:1263. doi: 10.1186/s12889-018-6117-y (PMC6238278; doi:10.1186/s12889-018-6117-y)
Supplement: Supplementary file 1 — Further demographic and background characteristics of survey participants, Description of data: Further demographic and background characteristics of survey participants. (DOCX 14 kb) [file 12889_2018_6117_MOESM1_ESM.docx]

**Additional file 1: Further demographic and background characteristics of survey participants**

| **Variable** | **Survey participants (N=232)**  **n (%)** |
| --- | --- |
| Adult | 135 (58.2) |
| Parent | 97 (41.8) |
| Education completed |  |
| Secondary only | 77 (33.2) |
| Further/Higher | 153 (65.9) |
| Employment status |  |
| Employed (full time) | 90 (38.8) |
| Employed (part time) | 65 (28.0) |
| Self-employed | 21 (9.0) |
| Not employed (not seeking employment- e.g. homemaker, retired, student) | 49 (21.1) |
| UK region of residence |  |
| East Midlands | 12 (5.2) |
| East of England | 10 (4.3) |
| London | 29 (12.5) |
| North East | 11 (4.7) |
| North West | 53 (22.8) |
| Northern Ireland | 3 (1.3) |
| Scotland | 10 (4.3) |
| South East | 30 (12.9) |
| South West | 15 (3.5) |
| Wales | 7 (3.0) |
| West Midlands | 15 (6.5) |
| Yorkshire & Humber | 36 (15.5) |

Where % total <100, there are missing values
