# Supplementary material for: Comparing the eating out experiences of consumers seeking to avoid different food allergens
Source: BMC Public Health. 2018 Nov 15;18:1263. doi: 10.1186/s12889-018-6117-y (PMC6238278; doi:10.1186/s12889-018-6117-y)
Supplement: Supplementary file 2 — Perceptions of information provision for participants avoiding Gluten (n = 149), Nuts (n = 272) and Milk (n = 77) prior to legislation, Description of data: Perceptions of information provision for participants avoiding Gluten (n = 149), Nuts (n = 272) and Milk (n = 77) prior to legislation. (DOCX 14 kb) [file 12889_2018_6117_MOESM2_ESM.docx]

**Additional file 2: Perceptions of information provision for participants avoiding Gluten (n=149), Nuts (n=272) and Milk (n=77) prior to legislation**

| **Survey subscale** | **Gluten** | **Nuts** | **Milk** |  |
| --- | --- | --- | --- | --- |
|  |  | **Mean (SD)** |  | **p** |
| Reliance on speaking to staff | 3.22 (1.48) | 3.75 (1.34) | 3.25 (1.38) | >.05 |
| Satisfaction with written information | 3.07 (0.97) | 3.14 (0.96) | 3.02 (1.07) | >.05 |
| Staff as an additional information source | 3.66 (1.21) | 3.90 (1.02) | 3.44 (1.18) | >.05 |
| Preference for a separate allergen menu | 3.98 (0.98) | 3.88 (1.04) | 3.63 (1.04) | >.05 |
| Menu invites you to ask staff about allergens | 4.71 (0.69) | 4.76 (0.57) | 4.56 (0.73) | >.05 |
| Sign invites you to ask staff about allergens^a^ | --- | --- | --- | --- |

^a^ Not measured prior to implementation of legislation
